# Syncope due to tracheal adenoid cystic carcinoma

**DOI:** 10.1002/rcr2.452

**Published:** 2019-07-02

**Authors:** Eva Marianne Theresa Bots, Abraham Christoffel van Wyk, Jacques Teran Janson, Riegardt Wagenaar, Gerald Paris, Coenraad Frederik Nicolaas Koegelenberg

**Affiliations:** ^1^ Division of Pulmonology Stellenbosch University Cape Town South Africa; ^2^ Pulmonology Department Erasmus Medical Centre Rotterdam the Netherlands; ^3^ Division of Anatomical Pathology, National Health Laboratory Service Stellenbosch University Cape Town South Africa; ^4^ Division of Cardiothoracic Surgery Stellenbosch University Cape Town South Africa; ^5^ Division of Radiation Oncology Stellenbosch University Cape Town South Africa

**Keywords:** Adenoid cystic carcinoma, radiotherapy, surgery, tracheal tumour

## Abstract

We present a case of a 34‐year‐old male who presented with syncope secondary to a large adenoid cystic carcinoma (ACC) of the distal trachea. A computed tomography and flexible bronchoscopy showed almost complete occlusion of the distal trachea. Resection with curative intent was performed, but resection margins were unfortunately not clear. The patient was subsequently offered adjuvant radiotherapy. Tracheal tumours comprise a small proportion of respiratory tract neoplasm, accounting for only about 2% of airway malignancies. Squamous cell carcinoma is the most common tracheal tumour, followed by ACC. Symptoms are usually attributable to the intraluminal component of the tumour causing an obstruction of the airway, resulting in stridor, dyspnoea, wheezing, haemoptysis, and cough. Syncope as a presenting symptom is exceedingly rare.

## Introduction

Tracheal tumours comprise a small proportion of respiratory tract neoplasms, accounting for only about 2% of airway malignancies 1. Squamous cell carcinoma is the most common malignant tracheal tumour, followed by adenoid cystic carcinoma (ACC) and other rare tumours such as mucoepidermoid carcinoma, lymphoma, sarcoma, and melanoma 2. ACC arises from glandular secretory cells, which are common in salivary glands. ACC is therefore most often found in the head and neck region but can also arise in the trachea, originating from submucosal seromucous glands 1, 3. Symptoms are usually attributable to the intraluminal component of the tumour causing an obstruction of the airway, potentially leading to stridor, dyspnoea, wheezing, haemoptysis, and cough 2. ACC is a slow‐growing malignant tumour with a deceptively bland histological appearance belying its high propensity for local recurrence. Three histological growth patterns have been described: tubular, solid, and cribriform. There is no association with smoking, and the distribution between genders is equal 2.

If the tumour is resectable, surgery is the first modality of choice. Incomplete resection is common 3, 4. In case of positive excision margins, adjuvant radiotherapy is recommended 4.

ACC has a good prognosis but is known for late local recurrence and metastasis for which long‐term follow up is indicated 4.

## Case Report

A 34‐year‐old male without significant medical history presented with a 4‐month history of shortness of breath and a non‐productive cough that worsened in the supine position. He eventually presented to a local district hospital with unexplained syncope.

**Figure 1:**
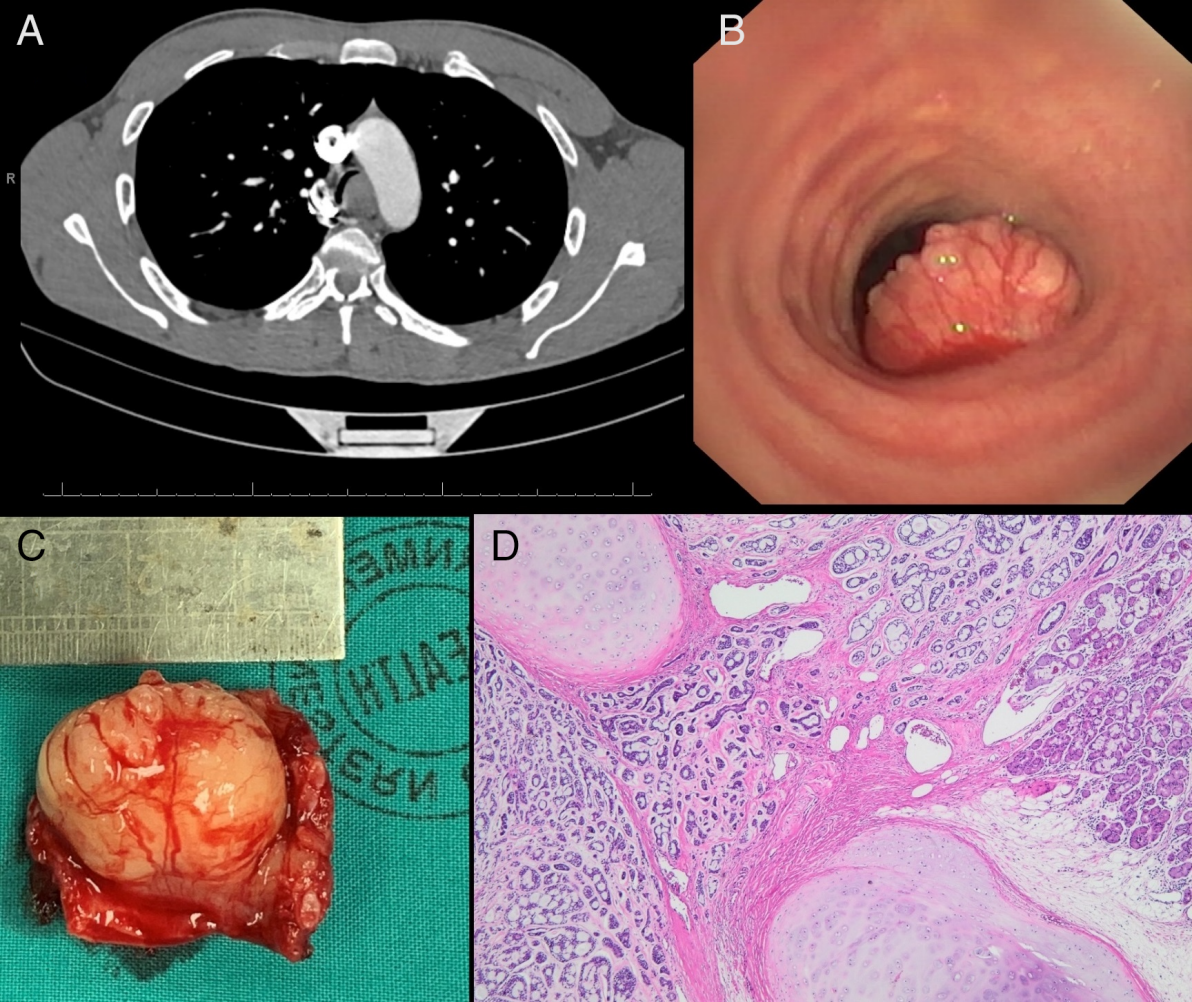
(A) The computed tomography scan showing almost complete obstruction of the distal trachea; (B) the endobronchial view seen on flexible bronchoscopy with the patient sitting—note the vascularity of the tumour; (C) the resected tumour; and (D) adenoid cystic carcinoma with a cribriform and tubular growth pattern infiltrates beyond the level of the tracheal cartilage into the peritracheal soft tissue (bottom left). Normal submucosal seromucous glands are present on the right (haematoxylin and eosin, original magnification 40×).

No clear cause for his symptoms was identified, and a computed tomography (CT) pulmonary angiogram (CTPA) was performed to rule out pulmonary embolism. No emboli were present, but the scan demonstrated a large intra‐tracheal tumour with near‐complete obstruction of the distal trachea (Fig. 1A).

The syncope was ascribed to likely transient asphyxiation due to intra‐tracheal obstruction, and the patient was referred to our unit. We proceeded to perform an urgent flexible bronchoscopy in the sitting position and confirmed a lobulated, smooth, solid, well‐vascularized mass that almost completely obstructed the distal trachea (Fig. 1B). Conventional fine‐needle aspiration did not yield diagnostic material (rapid on‐site evaluation), and forceps biopsies were subsequently cautiously obtained. Minimal bleeding occurred, and the histology of the biopsies showed a myoepithelial rich tumour with features favouring pleomorphic adenoma.

The patient was referred for resection of the tracheal tumour, and resection of the distal trachea at the carina and left main bronchus was performed (Fig. 1C). The patient had an uneventful postoperative course and was essentially asymptomatic at discharge. Histology of the resected specimen was in keeping with an ACC (Fig. 1D) that infiltrated through the cartilage into the peritracheal soft tissue. Two peritracheal and two subcarinal lymph nodes were negative for metastatic carcinoma.

**Figure 2:**
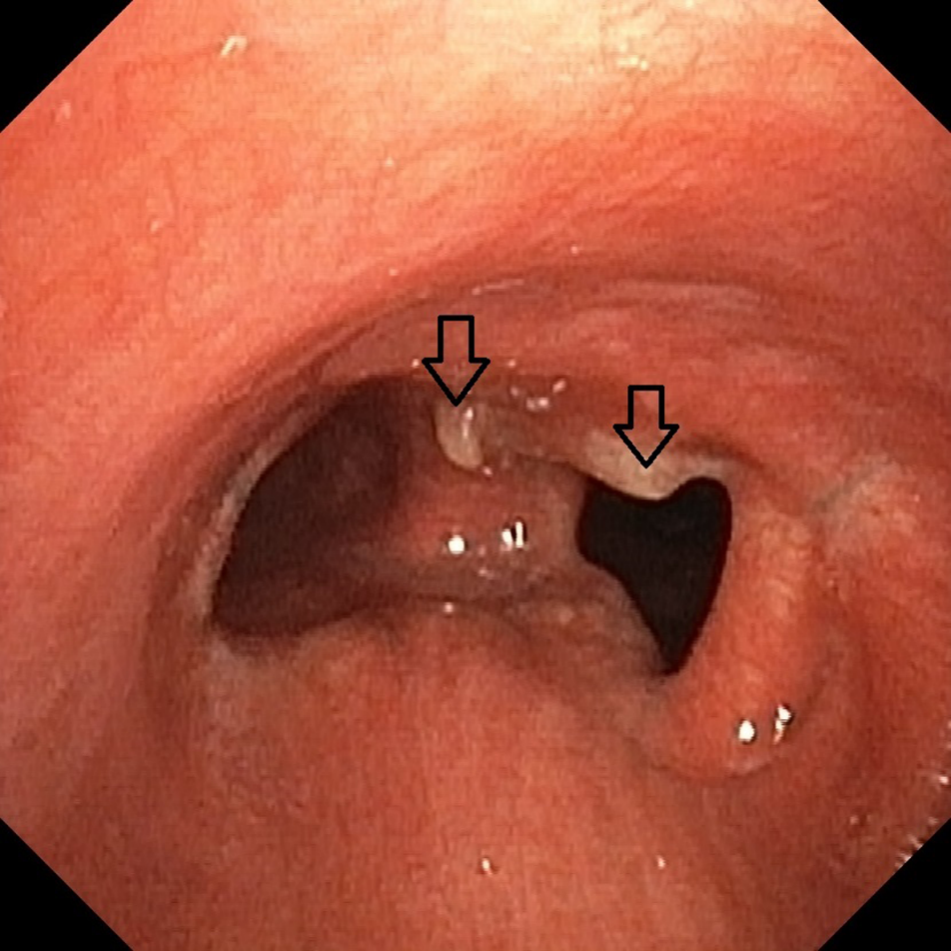
The surveillance bronchoscopy unfortunately showed local recurrence of the disease (arrows)

Tumour excision margins were unfortunately not clear of tumour infiltration on histology, and a surveillance bronchoscopy confirmed macroscopic recurrence of the primary tumour (Fig. 2). Although the differential diagnosis of these lesions included postoperative granulation tissue, the macroscopic appearance certainly favoured tumour recurrence (Fig. 2).

Our patient is currently undergoing adjuvant external beam radiotherapy, which may be followed by brachytherapy, if required, in a sufficient dose initially or for a later recurrence.

## Discussion

ACC is a rare malignant tracheal tumour that is characterized by slow growth. Symptoms are generally directly attributable to tracheal obstruction. Syncope, however, is not a common presenting complaint. A CT of the chest and bronchoscopy are the modalities of choice for pre‐resection evaluation and diagnosis 1.

The preferred primary treatment is surgical resection with clear margins and tracheal reconstruction when possible 2. The surgical technique for resection and reconstruction depends on the location within the trachea and the extent of tumour with or without positive lymph nodes 3, 4. In our case, a resection of the distal trachea and left main bronchus was performed due to the distal location of the ACC. Positive margins are common and have been reported in up to 15% of intramural and up to 85% of extramural cases 3.

Our patient's endobronchial biopsies showed a myoepithelial‐rich tumour with myxoid stroma and no clear histological features of malignancy, and therefore, pleomorphic adenoma was favoured. However, the resected specimen showed unequivocal invasion and a typical cribriform growth pattern of ACC. This highlights the diagnostic difficulty confronting the pathologist in diagnosing salivary gland‐type neoplasm on small biopsies. Many of the common salivary gland‐type neoplasms can show overlapping light microscopic and immunohistochemical features in limited biopsy material. ACC and pleomorphic adenoma share the combination of epithelial and myoepithelial cell differentiation. C‐KIT (CD117) has been reported to discriminate (positive in most ACCs) but only showed weak focal staining on the endobronchial biopsy, which was interpreted to negative 5.

ACC has a 5‐year overall survival rate of 50%, and prognosis is mainly determined by tumour margins and metastasis. If there is incomplete resection, adjuvant radiotherapy has been proven to prolong survival. There is no standard dosage. The unusual location of the tumour makes the approach to radiation therapy challenging. As in all cases, it is governed by the combined requirements of a high dose needed for a tumour of this type and the tolerances of the surrounding normal structures; here, most notably the heart and spinal cord as the other adjacent structures have a relatively high radiation tolerance. Brachytherapy will achieve a higher dose differential between the two but carries the risk of missing tumour cells due to its short effective range.

If the tracheal tumour is non‐resectable, radiotherapy is the modality of choice 1, 2. The 5‐year survival rate of resectable tracheal ACC has been reported to be up to 52%, with a 10‐year survival rate of 29%. In non‐resectable tumours, the 5‐year survival is 33%, and the 10‐ year survival rate is 10% 2.

### Disclosure Statement

Appropriate written informed consent was obtained for publication of this case report and accompanying images.
